# Dr. T. Gaillard Thomas

**Published:** 1863-08

**Authors:** 


Dr. T. Gaillard Thomas has lately been appointed Adjunct Professor of Obstetrics to the College of Physicians and Surgeons of this City. The choice of the Trustees is an exceedingly good one. Dr. Thomas is a gentleman of acknowledged ability in his department, and has long enjoyed the reputation of being a very successful teacher. We learn that Prof. Bedford has resigned the Chair of Midwifery in the University Medical College, which he has filled with so much ability since the first organization of the school. His successor has not been appointed. Prof. Wolcott Gibbs has been chosen to fill the Rumford Professorship, Harvard College, Mass. —[*American Medical Times.*

